# Blood Performance: A New Formula for Fish Growth and Health

**DOI:** 10.3390/biology10121236

**Published:** 2021-11-26

**Authors:** Moha Esmaeili

**Affiliations:** Institute for Marine and Antarctic Studies, University of Tasmania, Hobart Private Bag 49, 15-21 Nubeena Cres, Taroona, TAS 7053, Australia; moha.esmaeili@utas.edu.au

**Keywords:** haemoglobin, haematocrit, red blood cell, total protein, white blood cell

## Abstract

**Simple Summary:**

The use of haematological and blood biochemistry parameters has proven to be effective and repeatable ways to monitor fish health. Testing these parameters is becoming more common in aquaculture studies. Further, it is widely accepted that fish with better health status are more likely to grow faster as less energy should be consumed for non-growth purposes. Here, a new formula (Blood Performance) is introduced, which contains five common haematological and blood biochemistry parameters: red blood cells, white blood cells, haemoglobin, haematocrit, and total protein. The idea behind this formula is that any single component of this formula cannot be reliable enough as a biomarker of fish health and growth. However, interestingly, Blood Performance can be much more reliable and accurate for monitoring fish health and growth.

**Abstract:**

Monitoring fish health in a repeatable and accurate manner can contribute to the profitability and sustainability of aquaculture. Haematological and blood biochemistry parameters have been powerful tools and becoming increasingly common in aquaculture studies. Fish growth is closely related to its health status. A fish with a higher growth rate is more likely to be a healthy one. Any change in the physiological status of the fish, from pollution to nutritional stress, can cause changes in the blood parameters. Various aquaculture studies have measured the following components: red blood cells, white blood cells, haemoglobin, haematocrit, and total protein. However, because these parameters do not always follow the same trend across experimental fish, it is difficult to draw a firm conclusion about which parameter should be considered. Therefore, Blood Performance (BP) as a new formula is introduced, which is a more reliable indicator. This formula is simple and sums up the natural logarithm of the five above-mentioned parameters. More than 90 published peer-reviewed articles that measured these five parameters in the last six years confirmed the reliability and validity of this formula. Regardless of which supplements were added to the diets, the fish with a higher growth rate had higher BP as well. In addition, in 44 studies out of 53 articles, there was a significant positive correlation between specific growth rate and BP. Under different stressful situations, from pollution to thermal stress, the fish under stress had a lower BP than the control. Fish meal and fish oil replacement studies were further evidence for this formula and showed that adding excessive alternative proteins decreased growth along with BP. In conclusion, BP can be a reliable indicator of fish health and growth when it is compared between groups in the same experiment or farm. Although there was a positive correlation between specific growth rate and BP, comparing BP between experiments is not recommended. Standardising the haematological assays can improve the reliability and accuracy of BP across experiments.

## 1. Introduction

Aquaculture has been considered a sustainable option to provide food for humans. According to the latest statistics [1], there is an upward trend in the contribution of aquaculture in the total production of aquatic animals (45% in 2018), showing this food sector is on the right track for sustainability when compared to previous decades, when fish capture accounted for the majority of total production. However, encountering fish with stress in aquaculture systems is inevitable, and therefore, stress monitoring can be critical to the profitability and sustainability of aquaculture. There is a hierarchy change in the process of the stress response. Adrenalin is first produced from activation of the sympathetic nervous system, followed by glucose, and then lactate [2]. As a result, energy production is increased by stimulating glycogenolysis in the liver. Many parameters, such as plasma cortisol, glucose, lactate, and electrolyte concentrations, have been used for this purpose as primary and secondary stress responses [2]. Disturbances in plasma electrolytes occur only in stressful situations or under long-term stress [2].

Blood in fish transports a variety of constituents such as nutrients, hormones, minerals, immune components, microorganisms, water, gases, toxins, and waste products [3]. The most important functions of blood are the supply of oxygen and nutrients (including glucose, amino acids, and fatty acids) to cell tissues, removal of wastes (such as carbon dioxide, urea, and lactic acid), immunological functions, coagulation, and messenger functions [3]. Given the diverse critical roles of blood, measuring blood parameters may provide a more reliable picture of fish metabolism and health status. Haematology can provide useful information about fish welfare, health, immune system response, short-term and long-term effects of “suboptimal” farming conditions, water quality, potential disease outbreak, and nutritional status [4]. However, fish haematology is still not routinely measured in either research or farm conditions to assess health and/or welfare [4]. These aquaculture studies have measured red blood cells (RBC), white blood cells (WBC), haemoglobin (Hb), and haematocrit (Ht), as well as total protein (TP), as popular haematology parameters.

Haematological parameters and TP vary widely in different species, interindividual, and different farming conditions. For monitoring these variations, reference interval for different species in farming conditions or captured from the environment has been widely reported [5,6,7,8]. However, comparing haematology data as biological indicators between fish species or even the same species farmed in different experimental conditions is extremely difficult, if not impossible. Numerous factors, ranging from environmental variables to the sample collection process, have impacts on blood data, causing it to fall outside of the reference interval [9,10,11,12,13]. Despite these limitations, comparing haematological and blood biochemical parameters between treatments within the same experimental design can be a reliable monitoring tool.

In this research, a new parameter, Blood Performance (BP), that is the sum up RBC, WBC, Ht, Hb, and TP is introduced. It is believed that this parameter can better reflect the health of fish than any of the other parameters separately. Moha Esmaeili was the first to introduce an early version of this parameter [14], which has been applied in different fish [14,15,16,17,18], chicken [19,20], and turtle studies [21]. The focus of this review is on Teleostei, which includes the majority of aquaculture species. Three objectives were pursued: (i) monitoring these parameters where treatment resulted in significantly higher growth (HG) compared to control (S1) to find the possible connection between growth and BP, (ii) investigating these parameters in fish meal and oil (FM/FO) replacement studies where treatment resulted in no significant growth change compared to control or in cases where adverse effects by the inclusion of excessive alternative proteins or oils were observed. Finally, this formula was modified by adding a natural logarithm, and regression coefficients for this formula and correlation analysis between BP and its component (five parameters) were calculated to test their relations. Scatter plots were plotted to illustrate the relationship between BP with any single component (RBC, WBC, Ht, Hb, and TP) and specific growth rate (SGR). These plots show how the BP or its components are varied across experiments.

## 2. Literature Search, Regression Coefficients and Correlation Analysis

**Figure 1:**
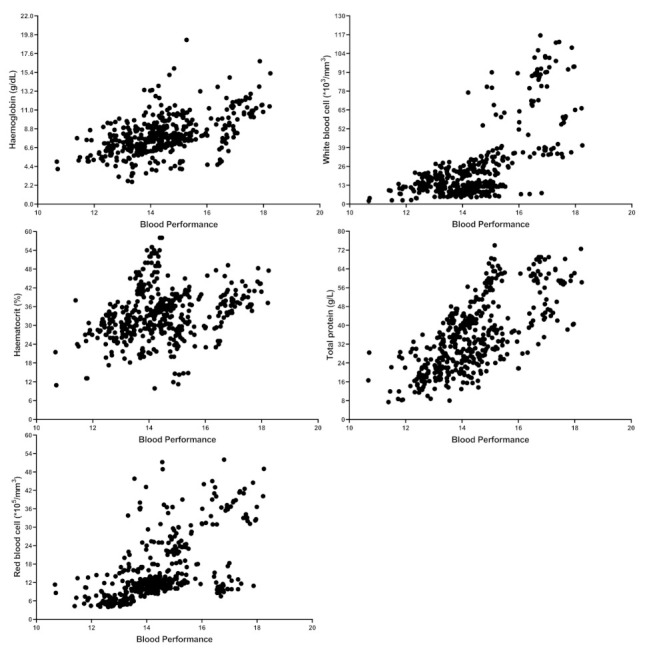
Distribution and relation between Blood Performance with haemoglobin, haematocrit, red blood cells, white blood cells and total protein. Each point indicates one sample from the raw data or published articles, and the number of samples is 441.

**Figure 2:**
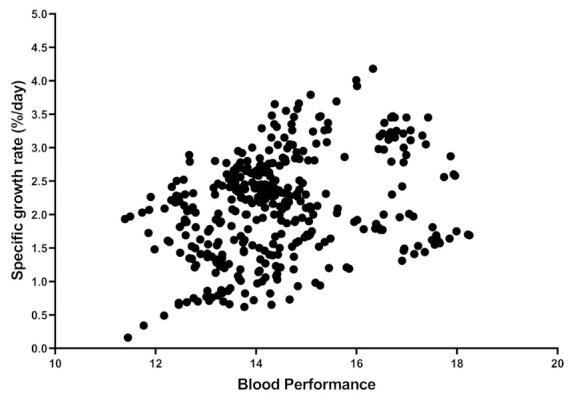
Distribution and relation between Blood Performance with specific growth rate. Each point indicates one sample from the raw data or published articles, and the number of samples is 380.

To find published articles with haematological parameters and TP, a systematic and comprehensive literature search in Google Scholar was conducted. After 2015, the search engine was set up with the keywords “RBC”, “WBC,” “mean corpuscular haemoglobin concentration: MCHC,” “aquaculture” and “TP”. The MCHC was searched as it includes both Hb and Ht. Subsequently, papers that were irrelevant or not peer-reviewed (not having Journal Citation Reports or Scientific Journal Rankings indexes) were deleted. To eliminate the effect of fish size, studies in juvenile size (the average for reviewed papers is around 41 g) and only studies in which the fish grew up at least 150% were selected. In the end, this review paper reported nearly 110 papers. Furthermore, data from published papers in Table S1 and shared raw datasets were collected to measure the contribution of each individual independent variable in the BP formula. As a result, over 441 samples were entered into the regression coefficients and correlation analysis (Table 1 and Table 2). The accuracy of the model could be improved if the authors could share more raw data. Control vs. HG fish (Table S1), control vs. stressed group (Table S2) and control vs. FM/FO replaced group (Table S3) for BP were considered for statistical analysis (independent sample *t*-Test). In doing so, two random numbers were generated based on the reported mean and standard deviation in published articles. While it would have been preferable to use real raw data from papers, this method is still defendable and did not affect the conclusion shown in the Supplementary Materials Tables. Figure 1 shows the scatter plot distribution of each variable against BP from 441 samples. Figure 2 shows the relationship between SGR and BP between 380 samples (from papers that include SGR data as well). All maximum, minimum, average, and other values were calculated from 441 different samples. SPSS software was used to perform the coefficient regression, correlation, and Independent Sample *t*-Test (Windows v21.0).

## 3. Haemoglobin

From a systematic standpoint to a deep physiological concept, Hb is one of the most studied proteins among biologists. The structure and diversity of this protein are beyond the scope of this paper and have been well described elsewhere [22]. Hb, which is measured using the cyanmethaemoglobin method, is in charge of aerobic metabolism, which involves reaching oxygen, dissolving large amounts of gas, and transporting it to the tissues. These gases are used by tissues as the final receptor of electrons derived from oxidative catabolic reactions and ATP metabolism [23]. Any stimulus, both internal and external, can influence metabolism, which in turn influences oxygen demand. As a result, the quantity and function of Hb play critical roles in basic metabolism and, ultimately, fish growth and health. This factor ranged from 19.9 to 2.6 g/dL in the current study, with an average of 7.98. Increasing Hb levels in the HG group in our previous studies after feeding fish with barberry root (*Berberis vulgaris*) [24] and garlic (*Allium sativum*) [25] were observed. Other studies showed a similar trend in HG fish when barramundi (*Lates calcarifer*) was fed on butyric acid [26], red hybrid tilapia (*Oreochromis mossambicus × O. niloticus*) on exopolysaccharide derived from reishi mushroom (*Ganoderma lucidum*) [27], and Siberian sturgeon (*Acipenser baerii*) on exogenous enzymes + probiotics [28] (Table S1). The increased concentration of Hb in the blood of these groups may have increased oxygen delivery to the tissues, and, as a result, their growth was improved. In FM/FO studies, treatments that resulted in no change or decrease in the growth compared to controls demonstrated the same trend in Hb levels. However, some studies found that Hb levels in HG fish did not change (Table S1). For instance, garlic in Caspian brown trout (*Salmo trutta caspius*) [29], carvacrol in tambaqui (*Colossoma macropomum*) [30], brewer’s yeast in Nile tilapia (*Oreochromis niloticus*) [31] and beluga (*Huso huso*) [32] enhanced growth but not Hb levels. These findings indicate that Hb cannot be used as a biomarker on its own. The possible reason can be the variability of energy demand and metabolism in different fish species and vertebrates [33,34], which eventually cause different oxygen demand and Hb. More research is needed to demonstrate the potential link between Hb and growth rate.

Stress causes an increase in oxygen demand in fish, which causes them to compensate with a variety of compensatory actions such as increased breathing frequency [35] or decreased synthesis of Hb (Table S2 and see review [36]). In the present review, the decrease of Hb with many different stressors such as ammonia [25,37], low temperature [38], microplastics [39], pollutions [40], pathogens [41,42], and heavy metals [43] were observed (Table S2). It seems that this decrease has been a global response to stress given that regardless of kind of stress and species, the Hb was decreased.

## 4. Haematocrit

The Ht (%) shows the volume of RBCs to the plasma. It is determined by microhaematocrit centrifugation, which sperate blood contents from plasma. It is widely accepted that higher Ht, showing higher viscosity, is beneficial for health [44]. In animals, from a long time ago, higher Ht is well connected to higher production [45]. This factor in the present review varied from 58.00 to 9.89 g/dL, and the average was 33.79 g/dL. In the present research, some connections between HG fish and Ht were observed. When fish fed a diet supplemented with purslane (*Portulaca oleracea*) [46], curcumin [47], bitter lemon (*Citrus limon*) [41], prebiotic inulin [48], thyroxine (T4 hormone) [49], macroalga (*Sargassum angustifolium*) [50], and selenium nanoparticulate [51], growth along with Ht value increased. The trend of growth and Ht in FM/FO studies was similar (Table S3). Conversely, formulating diets with garlic [25], ethylenediaminetetraacetic acid (EDTA) [15], butyric acid [26], benzoic acid [52], and lamb’s ears plant (*Stachys lavandulifolia*) [53] did not change Ht in HG fish. These inconsistencies demonstrate that Ht can be used as an indicator, but caution is required when drawing conclusions. However, an abnormally high level of Ht can indicate a variety of health issues, including dehydration and kidney disease [54]. Increasing Ht within a normal range can represent a good sign of optimised oxygen transport and health but not any unlimited increase.

With various kinds of stress in fish, the reduction of Ht was observed (Table S2). Ht is largely regulated with plasma levels. The increased plasma level (decrease Ht) enhances the electrolytes and protein movements through the blood flow to compensate for the oxygen demands of tissues. This parameter has dual but opposing effects on systemic oxygen transport (cardiac output and oxygen-carrying capacity). As a result, the relationship between oxygen transport and Ht is a parabolic shape [55].

## 5. Red Blood Cells

RBCs are the most common type of blood cells in vertebrates and are in charge of delivering oxygen (O_2_). RBCs absorb oxygen in the gills and release it into the tissues. The RBC count, which is measured with a Neubauer haemocytometer, is the number of RBCs per volume of blood. In fish, normal RBC counts range from 0.4 to 5.2, with an average of 1.56 (×10^6^/mm^3^) (Table S1). When compared to higher vertebrates, fishes have a lower number of RBCs per unit volume [56]. The study of 33 fish species revealed that the most active fishes (usually those at higher trophic levels) have a higher number of RBCs than the sluggish ones [56]. However, in the same experimental design using the same fish species and size, higher RBC can indicate potentially better oxygen delivery to tissues. The studies in peer-reviewed journals revealed that HG fish fed with various additives had a higher number of RBC. For example, ellagic acid in rainbow trout (*Oncorhynchus mykiss*) [57], brewer’s yeast in Nile tilapia [31], a combination of herbs in common carp (*Cyprinus carpio*) [58], butyric acid glycerides in yellowfin seabream (*Acanthopagrus latus*) [59], roselle anthocyanin extract in rainbow trout [60] and chitosan nanoparticles in Nile tilapia [43] increased the number of RBCs. Increased growth in these treatments demonstrates that fish benefit from optimised oxygen delivery, and there may be a direct relationship between growth and RBC numbers. However, when fish are fed on supplemented diets with EDTA [15], coriander (*Coriandrum sativum*) extract [61], β-glucan [62] and carvacrol [30], RBC did not change between control and HG fish. The growth and RBC trends in FM/FO studies were similar (Table S3). It demonstrates that this is not the case for all supplements and fish species and that multi-effects such as species and experimental conditions influence the growth-RBC relationship.

When fish are subjected to various stressors, RBC, unlike Hb and Ht, exhibits a variety of responses such as an increase, decrease, or no change (Table S2). For instance, when fish is exposed to ammonia stress [25], pathogens [63,64], feed restriction [65], and silver nanoparticles [66], no change or increase in RBC in the stressed group than control was observed. Oxidative stress (which is a result of external and internal stressors) can damage the RBC membrane and impair its deformability [67]. This shows that the quantity of the RBC is not the only factor, and it is possible that the shape of RBC is negatively changed by stress while the RBC number remains stable. However, in most cases, based on this review, this parameter was decreased, but more research is required to understand the response of fish RBC to stress.

## 6. White Blood Cells

WBCs are circulatory cells that help with both innate and acquired immune responses. Neutrophils (60–70% of total), eosinophils, basophils, lymphocytes, and monocytes constitute the different cell types. WBCs are counted in the Neubauer chamber using four marginal squares. The total WBC count was found to be related to body mass index [68]. The WBC ranged from 6.4 to 13.4 g/dL in 33 fish species [56] and 2.17 to 116.5 in our investigation (Table S1). Active fish had a lower number of WBC than less active ones, such as rohu (*Labeo rohita*) [56]. The higher number of WBC in HG fish fed on barberry root [24], garlic [25], ellagic acid [57], zeolite [69], butyric acid glycerides [59], prebiotic inulin [48] and selenium nanoparticulate [70] were observed. Conversely, no relation between growth and WBC was observed in different fish species when they were fed on roselle anthocyanin extract [60], exopolysaccharide [27] and coriander extract [61]. The growth and WBC trends in FM/FO studies were similar (Table S3). These findings can also be linked to immune system parameters, as WBC followed the same pattern as serum alternative complement activity (ACH50) and lysozyme. It is debatable whether the higher number of WBC was caused by the supplements or if it was simply a result of optimal health and growth regardless of additives.

Many, if not all, stress-related neuroendocrine elements influence immune response directly or indirectly [71]. Depending on their concentration, target cell, and the specific immune function studied, these elements have either an enhancing or suppressive effect on the immune system [71]. The increase of WBC under pollutions and herbicides [40,72], and silver nanoparticles [73], pathogens [63]; and no change with many other stressors [65,66,74,75] were observed. However, as with other haematological parameters, the majority of monitored studies revealed a decrease in WBC in the blood of stressed fish (Table S2).

## 7. Total Serum Protein

Total serum protein is one of the most common and useful blood parameters to measure. Serum proteins perform a wide range of functions, including maintaining osmotic pressure, pH, transporting various metabolites and interacting closely with the immune system. This parameter can show the nutritional status of the body indirectly [76]. TP plays an important role in fish humoral immunity and the innate immune response [77]. Commercial kits are the most used method for measuring TP in fish, and the values ranged from 0.74 to 7.5 (g/dL) with an average of 3.6 (g/dL) (Table S1). The articles showed feeding fish with garlic [25], curcumin [47], butyric acid glycerides [59], thyroxine [49], chitosan nanoparticles [78], macroalga [59] and encapsulated probiotic [79] increased TP in HG fish. On the other hand, TP was not increased in HG fish by some other additives, including barberry root [24], garlic [25], prebiotic inulin [48], exopolysaccharide [27], and sodium alginate + probiotic *P. acidilactici* [80]. The same relationship between TP and growth was observed in FM/FO studies (Table S3). As with the other parameters mentioned in the previous sections, an inconsistency in the literature was discovered, indicating that these parameters should be considered in conjunction with other factors. Furthermore, no positive relationship between TP and lysozyme and ACH50 was found, indicating that they are not strongly linked.

## 8. Blood Performance

This formula is based on the idea that any single component (Hb, Ht, RBC, WBC and TP) cannot be a reliable biomarker for fish growth or health. However, the BP may be a better choice because it considers all these variables in a formula. When a fish has a higher BP, it indicates that most of its variables were higher “overall,” but not always all. When we see variations in these five parameters across treatments, this formula makes more sense. The natural logarithm (Ln) was added to the formula to reduce the variation effect of variables. A review of the literature revealed a strong link between BP and growth (positive) and stress (negative). The positive correlation between SGR and BP (35%) in 380 samples showed that this parameter could be a reliable marker for growth. However, it should be noted that while BP is reliable and suitable for comparing treatments within each experiment, it is not suitable for comparing this parameter among experiments. Another issue is that many researchers believe that the higher value of these five parameters cannot be interpreted all times as a sign of better fish health. Based on previous experiences, in most cases, the high value of these parameters is a sign of better growth or health. This parameter ranged from 18.24 to 10.68, with 441 samples yielding an average of 14.43.

The BP formula is as follows:

Blood performance (BP) = Ln (Hb (g/dL) + Ln Ht (%) + Ln RBC (∗10^5^/mm^3^) + Ln WBC (∗10^3^/mm^3^) + Ln TP (g/L) (1)

### 8.1. Regression Coefficients and Correlation Analysis

Both standardised (SCR) and unstandardised regression coefficients (UCR) are useful for data interpretation. The UCR represents the amount by which the dependent variable (BP in the current study) changes if the independent variable is changed by one unit while the other independent variables (Hb, Ht, RBC, WBC and TP) remain constant. The SCR is measured in standard deviations and indicates how much a one-standard-deviation change in the independent variable results in a one-standard-deviation increase in the dependent variable. The SCR should be used when looking for independent variables that have a greater impact on the dependent variable. The UCR and *p*-value of coefficient regression both confirmed that all five parameters influence BP and that one unit change in BP results in 0.33, 0.28, 0.19, 0.15, and 0.08 change in TP, Ht, WBC, RBC, and Hb, respectively.

The SCR results confirmed that TP and WBC had the greatest impact on BP, while Hb had the least. The adjusted R square for this model was 0.956, indicating that these five parameters account for 95% of the change in BP. In general, the role of the immune system can be emphasised, as the most influential parameters were WBC and TP. BP correlates with all five parameters, indicating that they all play a role in this parameter. The highest correlation (68% and 67%) was found between BP and TP and between BP and WBC, while the lowest was found between Ht and BP (28%). Furthermore, TP was linked to RBC and Ht values. As previously reported in 33 fish species, there was no positive relationship between RBC and WBC [56]. Hb had a significant correlation with all parameters except TP; where correlation values were 35% (Ht), 21% (RBC), and 21% (WBC), respectively. Finally, the highest correlation was observed between SGR with BP (35%). The SGR had a correlation with Ht, and WBC as well.

### 8.2. Blood Performance and Growth

After absorbing energy from feed, this energy contributes to four major physiological components: growth, respiration, energy losses through faeces and nitrogenous excretory products [81]. If fish use less energy for non-growth purposes, they can save more energy for growth. When a fish is stressed, some energy is expended on maintaining and restoring the metabolism to normal. A proteomic study recently revealed that inefficient fish were stressed, and stress-related pathways such as proteolysis and response to ER stress were enriched [82]. Higher BP may indicate that tissues were well oxygenated due to an increase in Hb, Ht, and RBC. Furthermore, higher WBC and TP levels may have aided the immune system in fish with higher BP. These factors may explain the strong positive relationship between growth and BP.

A wide range of studies including supplementing diets with probiotics [28,48], herbal medicine [24,25,58], minerals [51,83,84], fatty acids [26,59,85], yeast [31,32], hormones [49], algae [50,86,87], polysaccharides [27,88] and lecithin [89] were covered to make a more solid conclusion about the relationship between growth and BP. In some of these results, at least four out of five parameters were increased and causing BP to be remarkably higher in the HG group. For instance, supplementing barberry root [24], purslane [46], curcumin [47], ellagic acid [57], and macroalga [50] to rainbow trout diets showed this trend. Similarly, zeolite in Snakehead murrel (*Channa striata*), lemon in ningu (*Labeo victorianus*) [41], butyric acid in yellowfin seabream [59], beet molasses in common carp [90], thyroxine hormone in sterlet sturgeon [49], chitosan nanoparticles in Nile tilapia [78] and probiotic in beluga [91] resulted in the same trend. These findings demonstrate how strongly BP is associated with growth, regardless of supplements or fish species from each trophic level. It should be noted that in this paper, only articles indexed in SJR and JCR after 2015 were tracked. The same pattern is most likely to be found in other studies. The main aim of this review is that to encourage other researchers to test this formula in their studies.

### 8.3. Blood Performance and Immune System (Lysozyme and ACH50)

As the first line of defence against pathogens, the fish body’s innate immune response protects it. The most important immune response molecules are monocytes/macrophages, neutrophils, non-specific cytotoxic cells, natural killer cells, mast cells, rodlet cells, complement, transferrins, interferon, anti-proteases, lysozyme, and C-reactive protein [92]. Among them, lysozyme and ACH50 have been commonly measured in aquaculture studies. When the fish diet was supplemented with barberry root [24], garlic [25], SBM + probiotic [14], purslane [46], curcumin [47], ellagic acid [57], zeolite [69], butyric acid [59] and probiotic [48] one or both parameters were changed in line with growth and BP (Table S1). In most cases, ACH50 and lysozyme levels increased with BP and growth. There was no increase in either growth or BP in FM/FO studies, and these two immune response parameters were not altered [93,94]. More research is needed to determine the growth-immune relationships and the role of BP in this process.

### 8.4. Blood Performance and Stress

Recent technological advances, as well as increased demand for fish production, have resulted in increased stock density in aquaculture. However, this condition exposes fish to chronic or, in some cases, acute stressors. The relationship between stress and immune function is extremely complex. Cortisol, in particular, disrupts the link between various immune system components and WBC production [95]. The details of how these pathways work is far from the scope of this research and well-reviewed elsewhere [96,97]. Glucose metabolism has a huge role in RBC metabolism as the cell energy is provided by glycolysis reaction and the pentose phosphate pathway [98]. Hb is a protein that can be found in RBCs and the lifespan of Hb is more than 100 days. As a result, any reaction in RBC can also affect Hb. Kosmachevskaya et al. (2021) thoroughly explained all involved proteins and mechanisms in Hb hemostasis. Unsurprisingly, the present literature search revealed that stress decreased BP and showed that this parameter can be a reliable indicator of stress (Table S2). Herbicides in snow trout (*Schizothorax plagiostomus*) and common carp [72,88,99], low-temperature stress in Malabar blood snapper (*Lutjanus molabaricus*) [38], microplastics in Nile Tilapia [39], ammonia in rockfish (*Sebastes schlegelii*) [37], *Aeromonas hydrophila* in ningu [41], reovirus in grass carp (*Ctenopharyngodon idella*) [42] and fungus toxin in common carp [100] stimulated the decrease of BP. Furthermore, the mean corpuscular volume (MCV), mean corpuscular haemoglobin (MCH) and MCHC fluctuated between studies showing that they cannot be reliable enough as a biomarker of stress. Although more research is needed, it can be claimed that BP was decreased in the majority of cases by a wide range of stressors in different fish species.

### 8.5. Blood Performance and Fish Meal/Oil Replacement

One of the most important areas of fish nutrition research was the FM/FO replacement studies. The future of aquaculture is heavily reliant on the discovery of alternative protein and oil resources. Despite the fact that the fish in these studies is farmed under ideal experimental conditions, nutritional stress impairs health and immunohaematological parameters. Growth is not the only important factor in FM/FO studies from a broad sustainable and long-term perspective; fish health should also be considered. During the farming period, a variety of stressors can have a negative impact on fish health. Therefore, if fish health is not optimum due to eating a “not ideal” diet, mortality and disease outbreak can threaten the system’s profitability. Interestingly, in the optimum replacement group, when there were no significant differences in growth compared to control, the BP followed the same trend. For example, Mata-Sotres et al. (2018) reported no negative effect on growth and BP when FO was replaced with a diet that contained PBM + Tallow + DHA. Amer et al. (2021) observed similar results when FM was replaced with insect meal (*Spodoptera littoralis*) up to 15% in diets. Further, no significant difference between growth and BP was observed when soybean meal was replaced up to 15% in California yellowtail (*Seriola dorsalis*) [101]. The soybean meal was totally replaced with leaf protein concentrate from carrot and sugar beet without impairment of growth and BP in Nile tilapia [102] (see more cases in Table S3). On the other hand, when an excessive amount of alternative proteins such as meat and bone meal [25,103], insect meal [94], and faba bean [104] replaced FM, the growth was declined along with BP. These results firstly show that haematological parameters can somewhat be reliable markers of health during FM/FO replacement process. More interestingly, BP followed the growth in both scenarios, showing that BP can be a better indicator when fish health is a concern. More research is required to validate the BP formula and connect it to other health parameters.

## 9. Conclusions and Prospect

Fish health and growth are closely related to each other, and healthy fish are more likely to grow faster. An increase in aquaculture activities and stock density to meet higher profitability subject fish to unavoidable stressful conditions. Monitoring these impacts can assist aquaculturists in avoiding massive mortality or negatively impacting fish welfare, health and growth. Immunohaematological parameters can be valid, repeatable and cost-effective ways to monitor fish health and growth. However, these parameters do not follow the same trend across treatments, making it difficult to draw a firm conclusion. They are similar to a package and monitoring them in a single formula can provide us with a more definitive conclusion. This paper describes and evaluates a new formula (BP) as an indicator of fish growth and health. This formula was found to be accurate in more than 90 peer-reviewed articles published in the last 6 years. In other words, it can be claimed that in more than 70% of cases, this formula works well, and fish with higher growth rates are more likely to have higher BP levels as well. Fish that were stressed for any reason, from pollutant to nutritional stress, had lower BP levels. The regression coefficient from over 441 samples confirmed that TP and WBC had the greatest impact on BP, emphasising the role of the immune system. The various units of the BP formula components, as well as differences between individuals and species, can be the limitations of this formula. Comparing BP across treatments in the same experiment, on the other hand, can provide a useful overview of the health and growth status. More reports are needed to validate this formula further, and it is recommended to compare it with other health parameters. Finally, the most important aim of the present preliminary review was that to encourage researchers to test this formula in their studies.

## Figures and Tables

**Table 1:** The result of coefficient regression with five independent variables (haemoglobin, haematocrit, red blood cell, white blood cell, total protein) to determine the portion of them in the Blood Performance formula. The sample size was 441 samples collected from literature reported in Table 1 and 10 complete raw datasets.

Variables	Unstandardised Coefficients	Standardised Coefficients			95.0% Confidence Interval	
	Beta	Beta	t	Sig.	Lower bound	Upper bound
Haemoglobin	0.079	0.074	4.839	0.000	0.057	0.930
Haematocrit	0.282	0.242	20.70	0.000	0.255	0.309
Red blood cell	0.146	0.278	23.11	0.000	0.134	0.159
White blood cell	0.195	0.518	48.67	0.000	0.187	0.203
Total protein	0.334	0.495	40.21	0.000	0.318	0.350

Dependent variable is Blood Performance. The R square of the model was 0.956 and significant (*p*-value = 0.00000). The stepwise method was tried as well, and the results showed the highest R square occurs when we include all five variables.

**Table 2:** Correlation analysis between Blood Performance and its components extracted from collected 410 sample data. The correlation between SGR and other parameters were in 380 sample data.

	Blood Performance	Ln Haemoglobin	Ln Haematocrit	Ln Red Blood Cell	Ln White Blood Cell	Ln Total Protein
Blood Performance	1					
Ln Haemoglobin	0.497 **	1				
Ln Haematocrit	0.282 **	0.346 **	1			
Ln Red blood cell	0.632 **	0.205 **	0.001	1		
Ln White blood cell	0.676 **	0.213 **	0.009	0.074	1	
Ln Total protein	0.667 **	0.104 *	0.255 **	0.471 **	0.161 *	1
Specific growth rate	0.35 **	0.054	0.252 **	0.051	0.284 **	0.188 *

* Correlation is significant at the 0.01 level (2-tailed). ** Correlation is significant at the 0.00005 level (2-tailed), which is corrected *p* value according to Bonferroni method.

## Data Availability

Data available on request due to privacy/ethical restrictions (the data that support the findings of this study are available on request from the corresponding author. The data are not publicly available due to privacy or ethical restrictions).

## Supplementary Materials

The following are available online at https://www.mdpi.com/article/10.3390/biology10121236/s1, Table S1: the relationship between supplementation to fish diet with growth, haematological parameters, blood performance, and immune system. Table S2: the relationship between supplementation to fish diet with growth, haematological parameters, blood performance, and immune system under different stressful situations. Table S3: the relationship between supplementation to fish diet with growth, haematological parameters, blood performance, and immune system in fish meal replacement studies. References: [105,106,107,108,109,110,111,112,113,114,115,116,117,118,119,120,121,122,123,124,125,126,127,128,129,130,131,132,133,134,135,136,137] are cited in supplementary materials.
