# Supplementary material for: Blood Performance: A New Formula for Fish Growth and Health
Source: Biology (Basel). 2021 Nov 26;10(12):1236. doi: 10.3390/biology10121236 (PMC8698978; doi:10.3390/biology10121236)
Supplement: Supplementary file 1 [file biology-10-01236-s001.zip › biology-1460368-supplementary.pdf]

Supplementary File Table S1. The relationship between supplementation to fish diet with growth, haematological parameters, blood performance, and immune system.

Supplement	Ha	Ha	Re	W	M	M	M	To	Bl	Gr	Ly	A	Species	Size	Comment	Refer
Barberry root	↑	↑	↑	↑	↓	_	↑	_	@	↑	↑	↑	Rainbow trout	16		[24]
Garlic	↑	__	↑	↑	__	__	__	__	↑	↑	↑	__	Rainbow trout	8		[25]
Purslane (1.5%)	↑	↑	↑	↑	_	↑	_	↑	↑	↑	↑	↑	Rainbow trout	3		[46]
Ashwagandha+ vit c (1%)	↑	↑	↑	↑	↓	↓	↓	↑	↑	@	_	@	Rohu	10	Mitigates the effect of low pH and waterborne iron toxicity	[107]
Curcumin (1%,2% 3%)	↑	↑	↑	↑	_	_	_	↑	↑	↑	↑	@	Rainbow trout	31	In all three dosages, improvement was observed	[47]
Ellagic acid	↑	↑	↑	↑	_	_	_	↑	↑	↑	↑	@	Rainbow trout	33		[57]
Zeolite (4%)	↑	↑	↑	↑	↑	↑	↑	↑	↑	↑	↑	↑	Snakehead murrel	34		[69]
Bitter lemon peels (5%)	↑	↑	↑	↑	↑	↑	↑	↑	↑	↑	↑	@	Ningu	21		[41]
Garlic	_	_	_	_	_	_	_	↑	↑	↑	↑	@	Brown trout	19		[29]
Brewer's yeast (1.5%)	_	_	↑	↑	_	_	_	_	↑	↑	@	@	Nile tilapia	6		[31]
Brewer's yeast	_	_	_	_	_	_	_	_	↑	↑	@	@	Beluga	11		[32]
(Oak acorn, coriander, and common mallow)	_	↑	↑	_	↑	↓	↓	↑	↑	↑	↑	↑	Common carp	20		[58]
Butyric acid glycerides (1%)	↑	↑	↑	↑	_	_	_	↑	↑	↑	↑	↑	Yellowfin seabream	9		[59]
Prebiotic inulin (15 g/kg)	↑	↑	↑	↑	↑	↑	↑	_	--	↑	↑	@	Asian seabass	7		[48]
EDTA (1.5%, 1% and 2%)	_	_	_	_	_	_	_	_	↑	@	@	@	Beluga	38	Mitigates the heavy metal stress	[15]
Beet molasses (2%)	↑	↑	↑	↑	_	_	_	↑	↑	@	↑	@	Common carps	20	Mitigates copper nano oxide stress	[90]
Roselle anthocyanin (1%)	↑	↑	↑	_	_	_	_	_	↑	_	↑	↑	Rainbow trout	14		[60]
Key lime and *bacillus licheniformis* (3%)	_	_	_	↑	_	_	_	↑	↑	↑	↑	@	Common carp	45		[118]
Butyric acid (5 g/kg)	↑	_	_	↑	↓	_	_	↑	↑	↑	↑	@	Barramundi	12		[26]
Exopolysaccharide	↑	↑	↑	_	_	_	_	_	↑	↑	@	@	Red hybrid tilapia	16		[27]
Ginger extract (0.2%)	↑	↑	↑	↑	_	_	_	↑	↑	↑	↑	↑	Common carp	11		[113]
Thyroxine t4	↑	↑	↑	↑	_	_	_	↑	↑	↑	@	@	Sterlet sturgeon	78		[49]
Coriander extract (2%)	↑	↑	_	_	_	_	_	_	↑	↑	↑	@	Rainbow trout	62		[61]
Exogenous enzymes + probiotics	↑	↑	↑	↓	_	_	_	↑	↑	↑	↑	@	Siberian sturgeon	138		[28]
Chitosan nanoparticles (0.5%)	↑	↑	↑	↑	↑	_	_	↑	↑	↑	@	@	Nile tilapia	6		[78]
Nanoparticulate Fu+Cu	↑	↑	↑	↑	↑	↑	_	↓	↑	↑	↑	↑	Snow trout	24		[84]
High carbohydrate + cinnamon	_	_	_	_	_	_	_	↓	↓	↓	@	@	Rainbow trout	16		[16]
Benzoic acid 0.1%	↑	_	_	_	_	_	_	_	↑	↑	@	@	Nile tilapia	6		[52]
Macroalga *Sargassum angustifolium*	↑	↑	↑	↑	_	↑	↑	↑	↑	↑	_	_	Rainbow trout	7		[50]
Bulk selenium and nano selenium 0.8 mg/kg	↑	↑	↑	↑	_	_	_	_	↑	↑	↑	@	Nile tilapia	5	both showed a same trend	[83]
Sodium Acetate +sodium propionate	↑	_	_	_	_	_	_	↑	↑	↑	↑	↑	Yellowfin seabream	6.5		[108,115]
wood betony extract (8%)	↑	_	_	↑	_	_	_	_	↑	↑	@	@	Rainbow trout	16		[53]

B-glucan	_	_	_	_	_	_	_	↑	↑	↑	↑	@	Nile tilapia	10		[62]
Triticale plant 20% in diet	_	_	_	_	_	_	_	_	_	_	_	@	Nile tilapia	14		[93]
Sodium alginate+ probiotic *p. Acidilactici*	_	_	↑	↑	_	_	_	_	↑	↑	↑	@	Asian sea bass	12		[80]
Olive leaf extract	↑	↑	↑	↑	_	_	_	↑	↑	↑	↑	@	Common carp	32		[114]
Vaccinated fish +probiotic *L. plantarum*	↑	_	_	↑	_	_	_	_	↑	↑	↑	↑	Rainbow trout	29		[117]
Carvacrol (1%)	_	_	_	↑	_	_	_	↑	↑	↑	@	@	Tambaqui	72		[30]
Common mallow (5%)	↑	↑	↑	↑	↑	↑	_	↑	↑	↑	↑	↑	Rainbow trout	10		[112]
Stinging nettle	↑	↑	↑	↑	@	@	@	↑	↑	↑	↑	@	Rainbow trout	42		[116]
Polysaccharide (fucoidan)	↑	↑	↑	↑	_	_	_	↑	↑	↑	@	@	Nile tilapia	17		[88]
Encapsulated probiotic (*G. candidum*)	↑	↑	↑	↑	↓	↓	↑	↑	↑	↑	@	@	Rohu	20		[79]
Nanoparticulate selenium	↑	↑	↑	↑	↑	_	_	↑	↑	↑	↑	@	Common carp	10		[51]
Prebiotic grobiotic®-a	↑	↑	↑	↑	_	_	_	↑	↑	↑	↑	@	Beluga	40		[91]
Soybean lecithin (6%)	↑	↑	↑	↑	_	↓	_	↑	↑	↑	↑	↑	Stellate sturgeon	11		[89]
Rose hip and safflower	_	_	_	↑	_	_	_	_	↑	↑	↑	↑	Beluga	26		[127]
n−3 LC-PUFA (1.2%)	↑	_	_	_	_	_	_	_	↑	↑	_	@	Silvery-black porgy	13		[128]
Probiotics *Enterococcus casseliflavus*	↑	↑	↑	↑	_	_	↑	↑	↑	↑	↑	@	Rainbow trout	38		[129]
Farming in 33 vs 20 degree	_	_	_	_	_	_	_	_	↑	↑	@	@	Asian sea bass	74		[123]
Tarragon	↓	↓	↓	↑	↓	↑	↑	↑	_	_	↑	@	Rainbow trout	7		[130]

MCV: mean corpuscular volume; MCH: mean corpuscular haemoglobin; MCHC: mean corpuscular haemoglobin concentration, ACH50: serum alternative complement activity. The upwards and downward arrows show the significant increase and decrease, respectively in the treatment with the highest growth as compared with control according to the Independent Samples T-Test (P<0.05). The minus sign shows no significant difference between control and treatment with the highest growth. For providing three replicates for analysis, two random numbers were generated with Excel based on the reported average and standard deviation. At sign (@) shows the parameter was not reported in the article.
Blood performance (BP)= Ln (Hb (g/dl) + Ln Ht (%) + Ln RBC (*10^5/mm^3) + Ln WBC (*10^3/mm^3) + Ln TP (g/l)

Supplementary File Table S2. The relationship between supplementation to fish diet with growth, haematological parameters, blood performance, and immune system under different stressful situations.

Supplement	Ha	Ha	Re	W	M	M	M	To	Bl	Ly	A	Species	Size	Comment	Refer
Ammonia stress	↓	_	_	↓	↑	↓	↓	↓	↓			Rainbow trout	8		[25]
Atrazine (4 ppm)	↓	↓	↓	↓	↑	↓	_	↓	↑	@	@	Snow trout	@		[106]
Low Temperature (22 vs 30)	↓	↓	↓	↓	_	_	_	_	↓	@	@	Snapper	167		[38]
Microplastics (15 Days, 10 mg/L)	↓	↓	↓	↓	↑	↑	↓	↑	↓	@	@	Nile tilapia	@		[39]
Silver nanoparticles	_	↓	↓	_	_	_	↑	↓	↓	↓	↓	Common carp	9	21 days, 50% of LC50	[66]
Praziquantel	↓	↓	↓	↑	_	_	_	↓	↓	@	@	African catfish	34		[40]
Silver nanoparticles (0.4 mg/l)	↓	↓	↑	↑	↓	↓	↑	↓	↓	@	@	Rainbow trout	250		[73]
Ammonia exposure in 19 c (1mg/l)	↓	↓	↓	↓	↓	↓	↓	↓	↓	@	@	Rockfish	38	4 Weeks	[37]
Aeromonas hydrophila	↓	↓	↓	↓	↓	↓	↓	↓	↓	↓	@	Ningu	21		[41]
Herbicides butachlor	↓	↓	↓	↑	_	_	_	↓	↓	@	@	Common carp	8		[72]
Herbicides oxadiazon	↓	↓	↓	↑	↑	↑	_	_	↓	@	@	Common carp	8		[72]

Aeromonas hydrophila	↓	↓	_	↑	_	↑	↓	↓	↓	↓	@	Goldfish	47		[63]
Endosulfan 96 h LC50	_	↓	_	↑	_	_	_	↓	↓	@	@	African catfish	65		[131]
Vibrio alginolyticus	_	_	_	_	↓	↓	↓	_	↓	_	@	Longtooth grouper	26		[64]
The LC50- 96h Crude Oil	↓	↑	↓	↓	↑	_	↓	↓	↓	@	@	Beluga	8		[105]
Clotrimazole 7.76 mg/l	↓	↓	↓	↓	↓	↑	↑	↓	↓	@	@	African catfish	27		[99]
Feed restriction	_	_	_	_	_	↓	↑	↑	↓	@	@	Siberian sturgeon	54	Decreased growth	[65]
Hg (0.13 ppm)	↓	↓	↓	_	_	_	_	↓	↓	↓	@	African catfish	80		[43]
UVA-induced	↓	_	↓	_	_	_	_	_	↓	@	@	African catfish	180	*Spirulina Platensis* mitigated stress	[75]
Polyethylene microparticles (5%)	_	↓	_	↓	_	_	_	_	↓	@	@	Rainbow trout	89		[109]
Atrazine herbicides	↓	↓	↓	↓	_	_	_	↓	↓	@	@	Nile tilapia	17	Fucoidan mitigated stress	[88]
Grass carp reovirus	↓	↓	↓	↓	_	_	_	↑	↓	@	@	Grass carp	25		[42]
Methyl mercury chloride toxicant	↓	_	↓	↓	_	_	_	↓	↓	@	@	Nile tilapia	23	Decreased growth	[70]
Fungal t-2 toxin	↓	↓	↓	↓	_	_	_		↓	@	@	Common carp	77		[100]
Phosalone (0.3 mg/l)	↓	↓	↓	↓	↑	↑	↑		↓	↓	@	Common carp	35		[132]
Pharmaceutical (chronic diclofenac)	↓	↓	↓	_	_	↑	↑	↓	↓	@	@	African catfish	30	1/16 96 Hours LC50	[74]
Nodavirus like-agent	↓	↓	↓	↓	↑	_	_	↓	↓	@	@	Mullet	75		[133]
Deltamethrin, 35 days	↓	↓	↓	_	↓	↓	↓	↓	↓	@	@	Major carp	7		[134]
NPK (15:15:15) fertiliser	↓	↓	↓	↑	↑	↓	↓	↓	_	@	@	African catfish	32		[135]
Uranyl acetate	↓	↓	_	↓	↓	↓	↓	↓	↓	@	@	African catfish	55		[136]

MCV: mean corpuscular volume; MCH: mean corpuscular haemoglobin; MCHC: mean corpuscular haemoglobin concentration, ACH50: serum alternative complement activity. The upwards and downward arrows show the significant increase and decrease, respectively, in the treatment under stress as compared with control according to the Independent Samples T-Test (P<0.05). The minus sign shows no significant difference between control and treatment under stress. For providing three replicates for analysis, two random numbers were generated with Excel based on the reported average and standard deviation. At sign (@) shows the parameter was not reported in the article.

Blood performance (BP)= Ln (Hb (g/dl) + Ln Ht (%) + Ln RBC ($*10^5/mm^3$) + Ln WBC ($*10^3/mm^3$) + Ln TP (g/l)

Supplementary File Table S3. The relationship between supplementation to fish diet with growth, haematological parameters, blood performance, and immune system in fish meal replacement studies.

Supplement	Ha	Ha	Re	W	M	M	M	To	Bl	Gr	Ly	A	Species	Size	Comment	Refer
MBM (65%)	↓	↓	↓	_	↓	_	↓	__	↓	↓	↓	↓	Rainbow trout	8	Garlic mitigates negative impacts	[25]
Insects mean (75%)	_	_	_	_	_	_	_	_	_	__	@	@	African catfish	4		[119]
Total replacement of SBM with plants	_	_	_	_	_	_	_	_	_	__	@	@	Nile tilapia	5	35% SBM in diet	[102]
70%SBM	_	_	↓	_	↑	↑	_	↓	↓	↓	↓	_	Beluga	351		[14]
70%SBM+probiotic	_	_	_	_	_	_	_	_	_	_	↑	↑	Beluga	351		[14]
Insect meal (20%)	_	_	_	_	_	_	_	_	↓	↓	__	@	Nile tilapia	17	Up to 15% no difference in growth and BP across treatments	[94]
Tallow, PBM+tallow+DHA	_	_	_	_	_	_	_	_	_	__	@	@	Totoaba	10		[85]
Macroalgae (*Sargassum ilicifolium*)	_	_	↑	↑	_	_	_	_	↑	↑	↑	↑	Barramundi	29		[86]
Spirulina platensis and *Chlorella vulgaris*	_	↑	↑	↑	_	_	_	↑	↑	↑	@	@	African catfish	42	Both increased	[87]
Soybean meal in diet 15%	_	_	_	_	_	_	_	_	_	_	@	@	California yellowtail	58		[101]
vegetable oils	_	_	_	_	_	_	_	_	_	_	@	@	African catfish	5		[124]

heat-treated soybean meal	↓	_	_	_	_	_	↓	_	_	_	@	_	Siberian sturgeon	190		[125]
50% unrefined peanut oil	_	↑	_	_	↑	_	↓	_	_	_	@	@	Seabream	10		[121]
Faba bean meal 10%	_	_	_	_	_	_	_	_	_	_	@	@	Beluga	82		[104]
Faba bean meal 25%	_	_	_	_	_	_	_	_	↓	↓	@	@	Beluga	82		[104]
Dry olive cake 12%	_	_	_	_	_	_	_	_	_	_	@	@	Hybrid tilapia	9		[122]
Jatropha platyphylla	_	↑	↑	_	_	↓	_	_	↑	_	@	@	Nile tilapia	14		[137]
Soybean meal	_	↑	↑	_	_	↓	_	_	↑	_	@	@	Nile tilapia	14		[137]
Fermented soy pulp 50%	↑	_	↑	_	_	↑	_	↑	_	↑	@	@	African catfish	8		[126]
Fermented soy pulp 100%	_	_	↓	_	_	_	_	↑	_	↓	@	@	African catfish	8		[126]
Yellow mealworm (50%)	_	↓	_	_	_		_	_	_	_	@	@	Rainbow trout	43		[120]

MCV: mean corpuscular volume; MCH: mean corpuscular haemoglobin; MCHC: mean corpuscular haemoglobin concentration, ACH50: serum alternative complement activity. The upwards and downward arrows show the significant increase and decrease, respectively, in the representative treatment as compared with control according to the Independent Samples T-Test ($P<0.05$). The minus sign shows no significant difference between control and t representative treatment. For providing three replicates for analysis, two random numbers were generated with Excel based on the reported average and standard deviation. At sign (@) shows the parameter was not reported in the article.

Blood performance (BP)= Ln (Hb (g/dl) + Ln Ht (%) + Ln RBC ($*10^5/mm^3$) + Ln WBC ($*10^3/mm^3$) + Ln TP (g/l)
